# Effect of CSA Concentration on the Ammonia Sensing Properties of CSA-Doped PA6/PANI Composite Nanofibers

**DOI:** 10.3390/s141121453

**Published:** 2014-11-13

**Authors:** Zengyuan Pang, Jiapeng Fu, Pengfei Lv, Fenglin Huang, Qufu Wei

**Affiliations:** Key Laboratory of Eco-Textiles, Ministry of Education, Jiangnan University, Wuxi 214122, Jiangsu, China; E-Mails: pangzengyuan1212@163.com (Z.P.); firgexiao@sina.cn (J.F.); vl1403979894@sina.com (P.L.); windhuang325@163.com (F.H.)

**Keywords:** polyaniline, camphor sulfonic acid, polyamide 6, ammonia, gas sensor

## Abstract

Camphor sulfonic acid (CSA)-doped polyamide 6/polyaniline (PA6/PANI) composite nanofibers were fabricated using *in situ* polymerization of aniline under different CSA concentrations (0.02, 0.04, 0.06, 0.08 and 0.10 M) with electrospun PA6 nanofibers as templates. The structural, morphological and ammonia sensing properties of the prepared composite nanofibers were studied using scanning electron microscopy (SEM), Fourier transform infrared spectroscopy (FT-IR), four-point probe techniques, X-ray diffraction (XRD) and a home-made gas sensing test system. All the results indicated that the CSA concentration had a great influence on the sensing properties of CSA-doped PA6/PANI composite nanofibers. The composite nanofibers doped with 0.02 M CSA showed the best ammonia sensing properties, with a significant sensitivity toward ammonia (NH_3_) at room temperature, superior to that of the composite nanofibers doped with 0.04–0.10 mol/L CSA. It was found that for high concentrations of CSA, the number of PANI–H^+^ reacted with NH_3_ would not make up a high proportion of all PANI–H^+^ within certain limits. As a result, within a certain range even though higher CSA-doped PA6/PANI nanofibers had better conductivity, their ammonia sensing performance would degrade.

## 1. Introduction

Gas sensors are important in environmental monitoring, home safety and chemical control [1]. Conducting polymers can work as gas sensors at room temperature [2,3], which is a competitive advantage compared with metal or metal oxides. As a result, conducting polymers have recently attracted more attention for realizing their applications in gas sensors [4,5]. Polyaniline (PANI) is a typical conducting polymer, having distinctive redox properties, good thermal stability [6,7], controllable conductivity and an easy fabrication process [8]. Due to these interesting properties, PANI has been a good potential candidate in batteries [9], electrochromic materials [10], electromagnetic shielding materials [11,12], metal anticorrosion products [13], tissue engineering [14] and sensing applications [15,16]. It is known that the conductivity of PANI can be influenced by some important factors, such as effective degree of polymerization, percentage of crystallinity, oxidation state level, percentage of doping, and type of dopant [17]. Depending on the oxidation states and doping processes, polyaniline has four forms: fully reduced leucoemeraldine base (LB), half oxidized emeraldine base (EB), half oxidized and protonated conducting emeraldine salt (ES), and fully oxidized pernigraniline base (PB) [18]. The ES state is highly conducting, while the others are mainly insulating in nature. By now, significant research efforts have been made to study the mechanism of doping or the effects of dopant on the properties of PANI. For example, Wang *et al.* [17] fabricated nanostructured PANI counter electrodes (CEs) using different H_2_SO_4_ doping concentrations for applications in dye-sensitized solar cells (DSSCs). It was found that PANI CE polymerized with 0.35 M H_2_SO_4_ shows the best photovoltaic performance with a solar-to-energy conversion efficiency of up to 5.57%. Arenas *et al.* [19] synthesized PANI with bicyclic aliphatic camphor sulfonic acid (CSA), aromatic toluenesulfonic acid (TSA) and carboxylic trifluoroacetic acid (TFA) were employed as dopants, and CSA mixed with TSA and CSA mixed with TFA were employed as the co-doping materials. The prepared PANI obtained from TSA-doping is the most sensitive in ammonia gas sensing. In addition, many researchers chose CSA as dopant. Patil *et al.* [1] prepared CSA-doped PANI-ZnO nanocomposites by grinding 10–50 wt.% of CSA into PANI-ZnO nanocomposite powder and then studied their ammonia sensing properties. Wu *et al.* [20] synthesized PANI by *in situ* polymerization of aniline with CSA as dopant, and then the sensors based on micro/nano-PANI were directly fabricated onto the interdigital electrodes by a self-assembly method. Zhou *et al.* [21] dissolved PANI and CSA into chloroform and *m*-cresol, and then the product was cast on slides to obtain CSA-PANI films. However, many of the materials mentioned above are powders, and for many functional materials, especially for those applied to sensors, specific surface area is very important. Nanofibers have larger specific surface area and their shapes are controllable. There are already several published papers dealing with the preparation of PANI-based nanofibers. Nevertheless, there are few reporting the effects of CSA concentration on the ammonia sensing properties of PANI-based composite nanofibers.

In this work, for the first time, CSA-doped PA6/PANI composite nanofibers were prepared as ammonia sensors by *in situ* polymerization of aniline under different CSA concentration (0.02, 0.04, 0.06, 0.08 and 0.10 M) conditions with electrospun PA6 nanofibers as templates. The ammonia sensing properties of the composite nanofibers and the sensing mechanism were studied and discussed.

## 2. Experimental Section

### 2.1. Materials

Polyamide 6 (PA6) (Mw = 2.1 × 10^4^ g/mol) was obtained from Zig Zheng Industrial Co. Ltd. (Taibei, Taiwan). Formic acid (FA), aniline monomer, ammonium persulfate (APS) and ammonium hydroxide (NH_3_·H_2_O) were purchased from Sinopharm Chemical Reagent Co. Ltd. (Beijing, China). (1*S*)-(+)-10-Camphorsulfonic acid (CSA) was purchased from Aladdin Reagent Database Inc. (Shanghai, China). All chemicals and reagents were used as received, except for aniline monomer, which was distilled twice under reduced pressure before use. Distilled water was used in this study.

### 2.2. Preparation of CSA-Doped PA6/PANI Composite Nanofibers

PA6 nanofibers were prepared by electrospinning PA6/FA solutions of 20 wt.% PA6 concentration. Firstly, aniline and APS were dissolved separately in CSA aqueous solutions, at a mole ratio of aniline to APS of 1:1. The CSA concentrations of the aqueous solutions were 0.02, 0.04, 0.06, 0.08 and 0.10 M, respectively. Secondly, the electrospun PA6 nanofibers were immersed into the aniline/CSA solution for 30 min. Then, polymerization of aniline was initiated by dropping the APS/CSA solution into the above diffusion bath. PANI was synthesized on the surface of PA6 nanofibers and doped with CSA at 0–5 °C for 5 h. Finally, the samples were taken out and the rest of the PANI in the bath was also collected by filtration. All composite nanofibers and CSA-PANI nanopowders (490–620 nm) were washed with deionized water followed by acetone, and dried in vacuum at 50 °C for 24 h.

### 2.3. Characterization

The surface morphologies of the CSA-doped PA6/PANI composite nanofibers were investigated using a field emission scanning electron microscope (FESEM, S-4800, Hitachi, Tokyo, Japan). Fourier transform infrared (FTIR) spectra were obtained in the range of 4000–400 cm^−1^ with a 4 cm^−1^ spectral resolution by using a NEXUS 470 spectrometer (Nicolet, Madison, WI, USA). The conductive properties of the composite nanofibers and composite nanopowders were investigated by four-point probe techniques. The crystalline structure analysis was also performed on a D8 Advance X-diffractometer (Bruker AXS, Billerica, Germany), over the 2θ range of 3°–90°. The particle diameter was measured by Brookhaven Instruments (Zeta Plus, Holtsville, NY, USA).

### 2.4. Ammonia Sensing Tests

**Figure 1.**
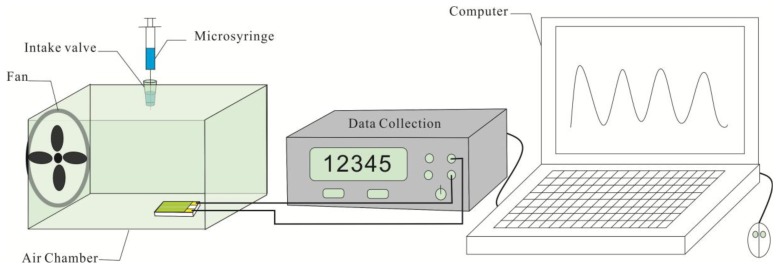
Schematic illustration of home-made gas sensing system.

The sensing behaviors of the samples to NH_3_ were investigated with a home-made test system as shown in Figure 1. A home-made Au electrode with a gap of 0.5 mm between two Au stripes was firstly prepared by depositing Au on phenolic resin, and then the prepared CSA-PA6/PANI composite nanofibers with area of 4 × 4 mm^2^ were pasted onto the open area between the two electrodes. The tests were performed at room temperature (25 ± 1 °C). The actual ammonia volumes injected into the air chamber were 0.3367, 0.6734, 1.3468, 2.0202, 2.6936 and 3.3670 μL, corresponding to ammonia vapor with the concentration of 25, 50, 100, 150, 200 and 250 ppm respectively. The resistance of the composite nanofbiers was measured using an Agilent-34401A data acquisition system (Agilent Technologies (China) Co. Ltd., Beijing, China). The resistance of CSA-doped PA6/PANI composite nanofibers in air (R_0_) and in the presence of NH_3_ (R_i_) were measured to evaluate the gas response, S, defined as S(%) = (R_i_ − R_0_)/R_0_ × 100.

## 3. Results and Discussion

### 3.1. Surface Morphology

**Figure 2.**
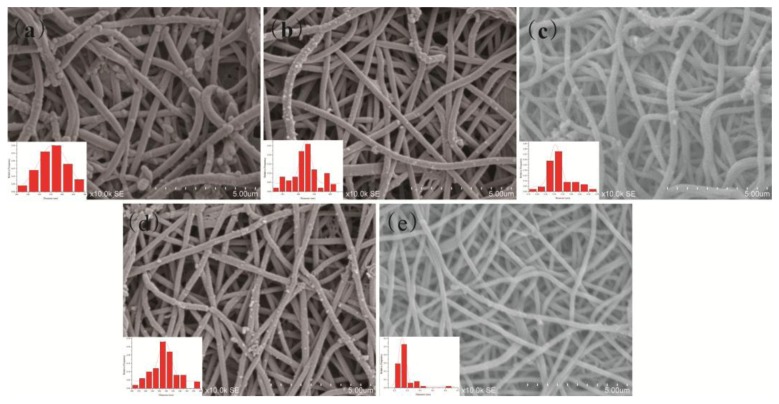
SEM images and diameter distributions of (**a**) 0.02 M (**b**) 0.04 M (**c**) 0.06 M (**d**) 0.08 M (**e**) 0.10 M CSA-PA6/PANI composite nanofibers.

Figure 2 shows the SEM images and diameter distributions of CSA-doped PA6/PANI composite nanofibers with different CSA concentrations of 0.02, 0.04, 0.06, 0.08 and 0.10 M, denoted respectively as 0.02 M CSA-PA6/PANI, 0.04 M CSA-PA6/PANI, 0.06 M CSA-PA6/PANI, 0.08 M CSA-PA6/PANI and 0.10 M CSA-PA6/PANI. The SEM images for all PA6/PANI composite nanofibers doped with CSA indicated a non-woven film with good fiber structure.

For PANI, fiber structure would provide a big specific surface area which could help improve the sensitivity of a gas sensor [22]. The surfaces of the composite nanofibers were not very smooth, with some particles on the surface. The particles may be some residual PANI. The diameter distributions showed that the diameter of 0.02 M CSA-PA6/PANI appeared more uniform, and the average diameters of the composite nanofibers, which was around 320 nm looked similar.

### 3.2. FTIR Analysis

**Figure 3.**
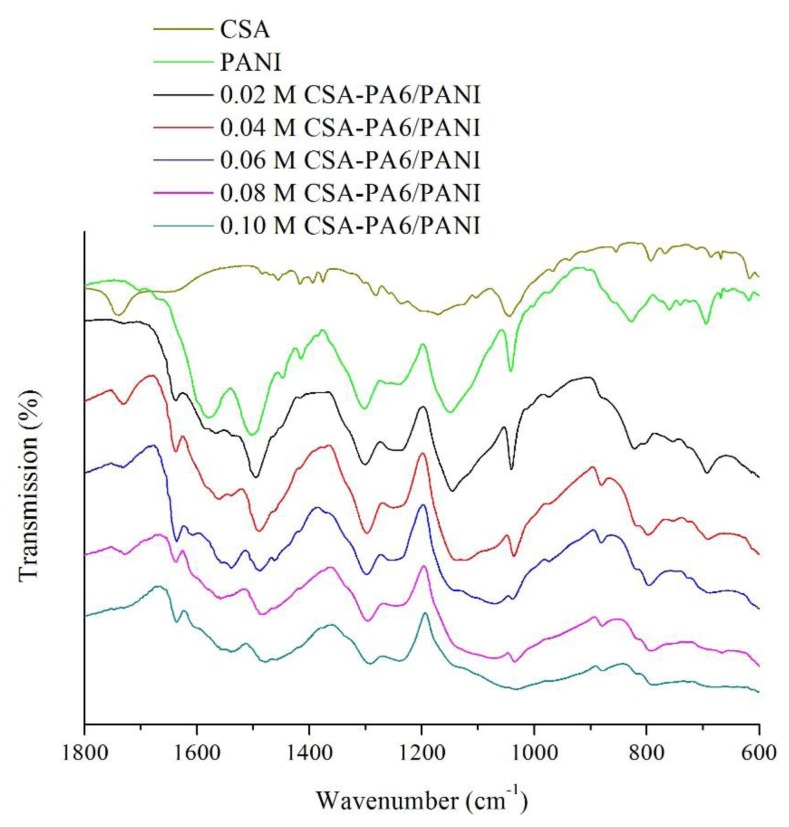
FTIR spectra of the pure CSA, pure PANI and the prepared CSA-PA6/PANI composite nanofibers.

Figure 3 presents the FTIR spectra of the pure CSA, pure PANI and the prepared CSA-PA6/PANI composite nanofibers. It was observed that the characteristic peak of CSA around 1740 cm^−1^ [23] can be found at the other spectra of CSA-PA6/PANI composite nanofibers. For pure PANI, the band at 1495 cm^−1^ assigned to the stretching vibration of the C-C in the benzene ring and the peak around 1559 cm^−1^ originated from stretching vibrations of C=N and C=C bonds in quinone units can be found. These characteristic peaks also existed at the spectra of the CSA-PA6/PANI composite nanofibers. In addition, the stretching vibration peak at 1637 cm^−1^ assigned to the C=O stretching of PA6 can be found at the spectra of the CSA-PA6/PANI composite nanofibers, too. It can also be observed that the characteristic peaks of the prepared composite nanofibers shifted slightly. This may be the results of the differences in doping percentage.

### 3.3. XRD Patterns

**Figure 4.**
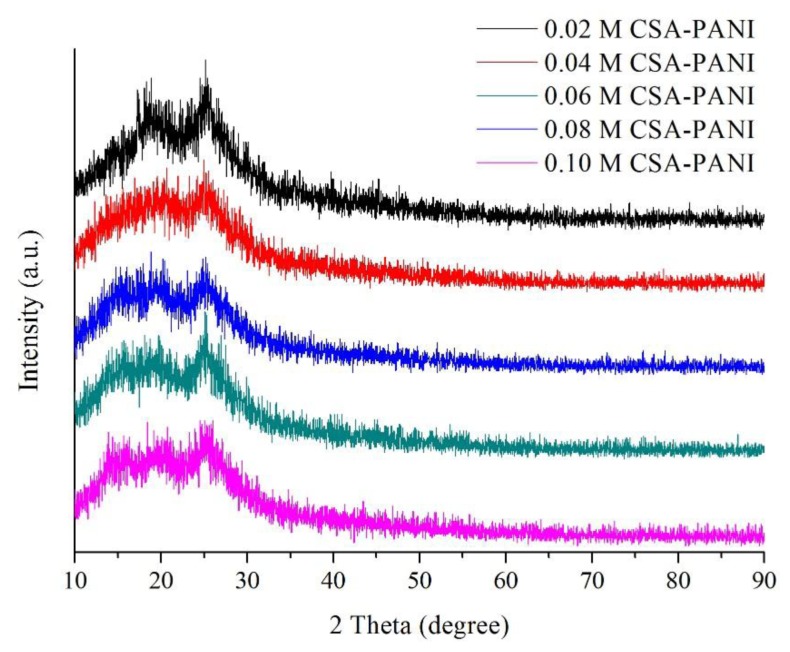
X-ray diffraction patterns of the prepared CSA-PANI nanopowders.

The X-ray diffraction patterns of CSA-PANI nanopowders with different doping degrees are presented in Figure 4. It is known that the characteristic peaks of PANI are at 2θ = 16°, 21° and 26°. The characteristic peaks of PANI can be seen clearly. In addition, with increase of concentration, more and more sharp peaks are observed, indicating that the crystallinity of CSA was increased. However, when the doping concentration was too high, CSA could not be completely doped into PANI and had to adhere on the surface of the film [21].

### 3.4. Conductive Properties

**Figure 5.**
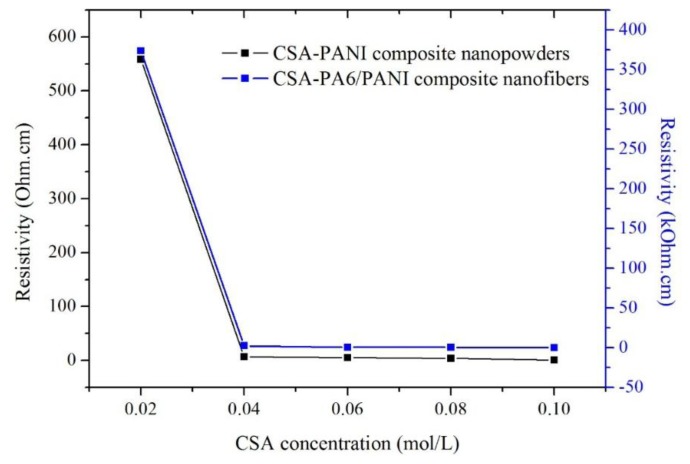
The resistance of CSA-PANI nanopowders and CSA-PA6/PANI composite nanofibers.

Figure 5 shows the resistivity of the CSA-PANI nanopowders and CSA-PA6/PANI composite nanofibers in air at room temperature. The prepared materials doped with 0.02 M CSA showed the highest resistivity and the conductivities of those doped with 0.10 M CSA were the best. In addition, the conductivities of the prepared materials doped with 0.02 M CSA were very different from those of the others doped with higher CSA concentrations.

This showed that when the CSA concentration was in the range of 0.02–0.10 M, the higher the CSA concentration was, the better conduction the materials had, which was because there were more H^+^ captured by PANI chains. However, the sensitivity of the prepared nanofibers did not become higher when the nanofibers' conduction was improved.

### 3.5. Ammonia Sensing Properties

#### 3.5.1. Gas Sensitivity of CSA-Doped PA6/PANI Composite Nanofibers

**Figure 6.**
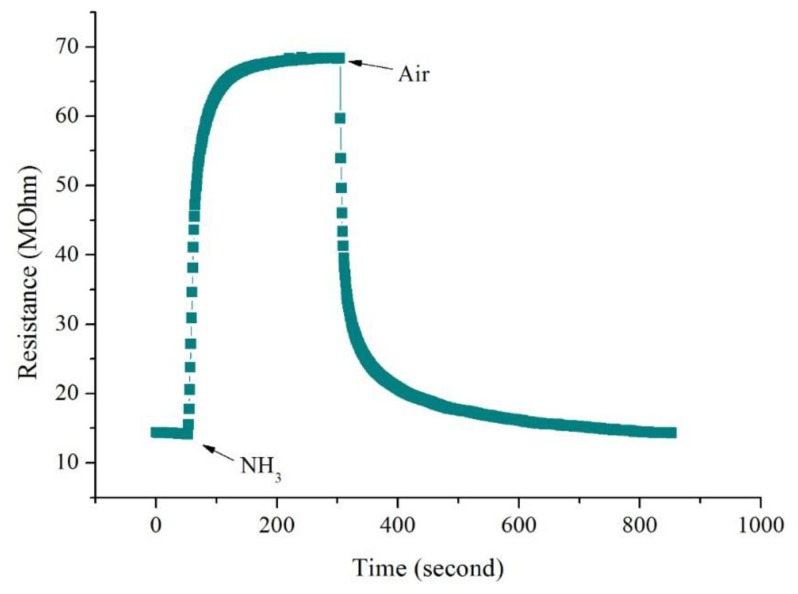
Changes in resistance of the CSA-doped PA6/PANI composite nanofibers with respect to time on the exposure of NH_3_ gas.

The changes in resistance of CSA-doped PA6/PANI composite nanofibers with respect to time on the exposure of NH_3_ are shown in Figure 6. It is obvious that the resistance of the CSA-doped PA6/PANI composite nanofibers increased dramatically when they were exposed to NH_3_. A moment later, the resistance became stable. When the gas room was opened and fresh air was introduced, the resistance decreased and returned to the original resistance gradually.

**Figure 7.**
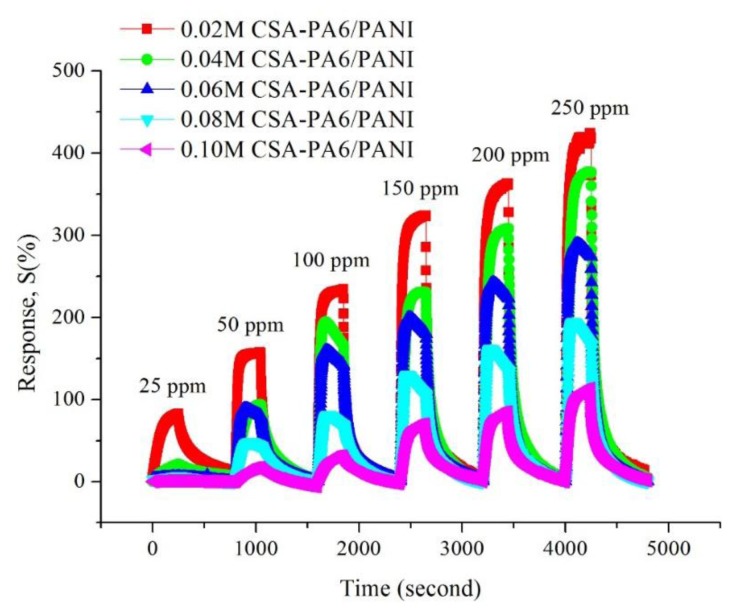
Dynamic response of the CSA-doped PA6/PANI composite nanofibers to 25–250 ppm NH_3_ at room temperature.

**Figure 8.**
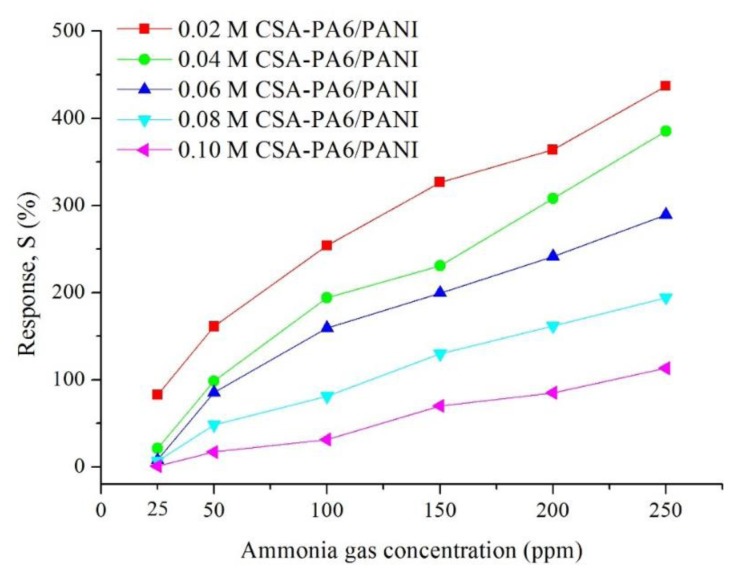
Response of the CSA-doped PA6/PANI composite nanofibers for 25–250 ppm NH_3_.

Figure 7 shows the gas response of CSA (0.02–0.10 M)-doped PA6/PANI composite nanofibers for 25–250 ppm NH_3_ gas. The response of CSA-doped PA6/PANI composite nanofibers was enhanced with increasing concentration of NH_3_ gas from 25 to 250 ppm operating at room temperature. The CSA-doped PA6/PANI composite nanofibers gas sensors all showed a good sensitivity to ammonia vapor, and the gas sensor of 0.02 M CSA-doped PA6/PANI composite nanofibers possessed the highest response amplitude to ammonia among the five sensors tested. The response values are depicted in Figure 8 which is obtained from Figure 7. It is very clear that the response of 0.02 M CSA-doped PA6/PANI composite nanofibers sensor was much higher than the other four composite nanofibers sensors. The response value of 0.02 M CSA-doped PA6/PANI composite nanofibers sensor to 250 ppm ammonia was 436.98% which was 1.13, 1.51, 2.25 and 3.86 times of that of 0.04 M, 0.06 M, 0.08 M and 0.10 M CSA-doped PA6/PANI composite nanofibers sensors, respectively. When the NH_3_ concentration was 100 ppm, the response value of 0.02 M CSA-PA6/PANI was 253.96%. This was superior to the results (100.00%, 215.60% and 91.00%) recently reported by Pang *et al.* [24], Pan *et al.* [25] and Khuspe *et al.* [26].

#### 3.5.2. Gas Sensitivity of 0.02 M CSA-Doped PA6/PANI Composite Nanofibers

**Figure 9.**
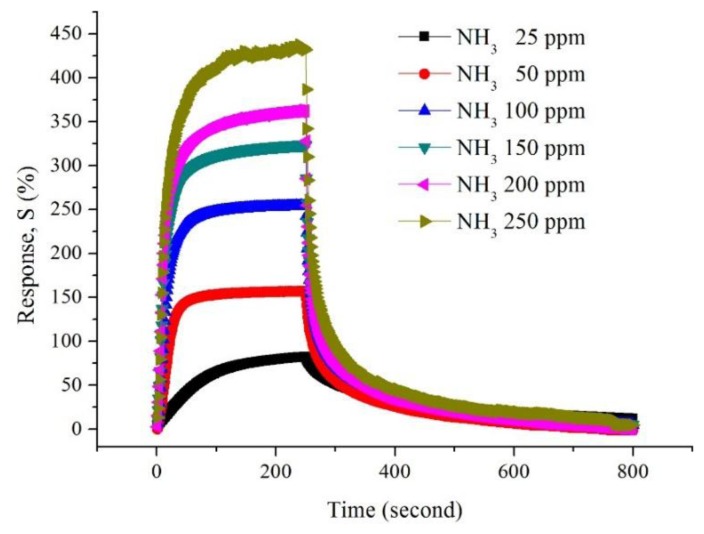
Response of 0.02 M CSA-doped PA6/PANI composite nanofibers to 25–250 ppm NH_3_.

**Figure 10.**
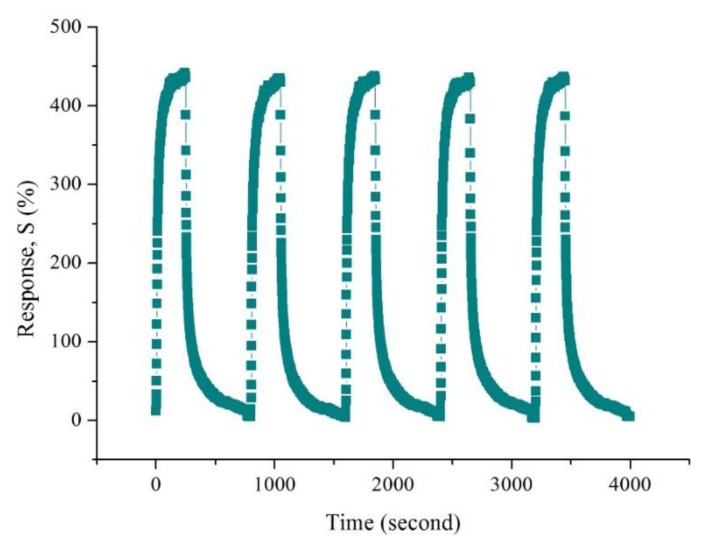
Repeatability of 0.02 M CSA-doped PA6/PANI composite nanofibers to 250 ppm NH_3_.

Because the 0.02 M CSA-doped PA6/PANI composite nanofibers gas sensor showed the highest sensitivity among the samples tested, the other properties of 0.02 M CSA-doped PA6/PANI composite nanofibers were also examined. Figure 9 shows the response of 0.02 M CSA-doped PA6/PANI composite nanofibers to 25–250 ppm of NH_3_. The sensitivity of the composite nanofibers increased with increasing concentration of NH_3_ gas from 25 to 250 ppm and it was found to be 82.67%, 161.16%, 253.96%, 326.54%, 363.90%, 436.98%, respectively operating at room temperature. The repeatability is a very important property of gas sensor. The response of 0.02 M CSA-doped PA6/PANI composite nanofibers gas sensor was monitored for repeated exposure and removal of 250 ppm ammonia up to five cycles, as shown in Figure 10.

**Figure 11.**
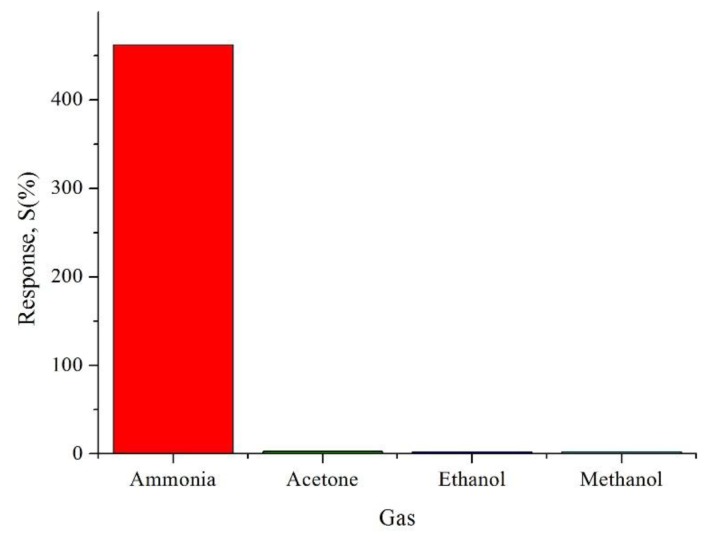
Sensor response of 0.02 M CSA-doped PA6/PANI composite nanofibers to 250 ppm different gases.

It can be observed that the prepared CSA-doped PA6/PANI composite nanofibers sensor had high repeatability. In addition, to test the cross sensitivity, 0.02 M CSA-doped PA6/PANI composite nanofibers were exposed to 250 ppm acetone, ethanol and methanol, respectively, at room temperature. As shown in Figure 11, it can be seen that there was a distinct difference in the response to the tested gases. The sensors showed very weak responses to acetone, ethanol and methanol. As a result, it can be concluded that the sensor exhibited high selectivity to NH_3_.

#### 3.5.3. Sensing Mechanism of CSA-Doped PA6/PANI Composite Nanofibers

**Figure 12.**
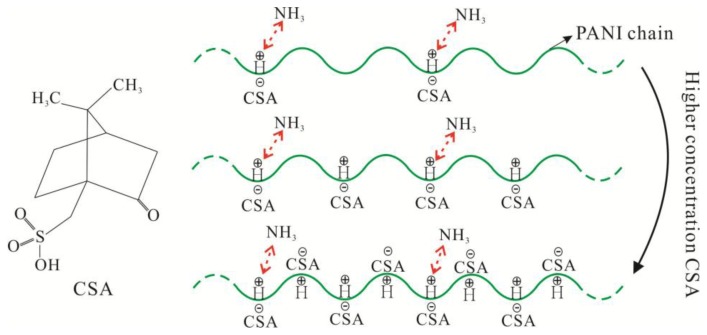
Schematic sensing mechanism of CSA-doped PA6/PANI composite nanofibers.

The sensing mechanism of CSA-doped PA6/PANI composite nanofibers is illustrated in Figure 12. As shown in Figure 12, PANI was doped with CSA, which was a protonic acid with large molecules.

When exposed to NH_3_, PANI was de-doped and the reversible reaction [27]: PANI–H^+^ + NH_3_ ⇌ PANI + NH^4+^ ocurred. As a result, the resistance of PANI changed, embodied as sensitivity. To improve the conductivity of PANI, high concentrations of CSA were introduced, within certain limits. High concentrations of CSA would improve the conductivity of PANI and more PANI–H^+^ would exist. When the NH_3_ concentration was constant, the number of NH_3_ molecules was also constant under certain conditions. This resulted in a certain amount of the PANI–H^+^ which could react with NH_3_. Then, when the concentration of CSA was high, the PANI–H^+^ reacted with NH_3_ would not make up a high proportion of all PANI–H^+^ within certain limits. As a result, the resistance change of the tested sensor would not account for a big percentage of the initial resistance. That is, the value of S(%) = (R_i_ − R_0_)/R_0_ × 100 was not very obvious, which appeared as low sensitivity. In addition, when the large molecule protonic acid CSA modified the PA6/PANI composite nanofibers as a dopant, it would increase the steric hindrance of the de-doping reactions. Therefore, when the concentration of CSA increased, the response values of the composite nanofibers decreased.

## 4. Conclusions

The present work reported the preparation of different concentration CSA-doped PA6/PANI composite nanofibers for the detection of NH_3_ gas at room temperature. The gas sensing properties of the composite nanofibers to NH_3_ indicated that these thin films of CSA-doped PA6/PANI composite nanofibers were good candidates for NH_3_ detection. It was also found that for high concentrations of CSA, the number of the PANI–H^+^ reacted with NH_3_ would not make up a high proportion of all PANI–H^+^ within certain limits and steric hindrance would reduce NH_3_'s chances of being absorbed by PANI. As a result, even though high concentration CSA-doped PA6/PANI nanofibers had better conductivity, their ammonia sensing performance was degraded.
